# A Chromosome-Level Reference Assembly and Annotation for the White-Faced Plover (*Charadrius dealbatus*) Genome

**DOI:** 10.1093/gbe/evaf219

**Published:** 2025-12-03

**Authors:** Ying Zeng, Jie Tu, Heiman Ho, Xiaotong Niu, Hongzhou Lin, Yachang Cheng, Zitan Song, Chenqing Zheng, Yang Liu

**Affiliations:** State Key Laboratory of Biocontrol, School of Ecology, Sun Yat-sen University, Shenzhen 518107, China; The Roslin Institute and Royal (Dick) School of Veterinary Studies R(D)SVS, University of Edinburgh, Easter Bush Campus, Midlothian EH25 9RG, UK; State Key Laboratory of Biocontrol, School of Ecology, Sun Yat-sen University, Shenzhen 518107, China; State Key Laboratory of Biocontrol, School of Ecology, Sun Yat-sen University, Shenzhen 518107, China; State Key Laboratory of Biocontrol, School of Ecology, Sun Yat-sen University, Shenzhen 518107, China; State Key Laboratory of Biocontrol, School of Ecology, Sun Yat-sen University, Shenzhen 518107, China; State Key Laboratory of Biocontrol, School of Ecology, Sun Yat-sen University, Shenzhen 518107, China; State Key Laboratory of Biocontrol, School of Ecology, Sun Yat-sen University, Shenzhen 518107, China; Comparative Socioecology Group, Department for the Ecology of Animal Societies, Max Planck Institute of Animal Behavior, Konstanz 78467, Germany; State Key Laboratory of Biocontrol, School of Ecology, Sun Yat-sen University, Shenzhen 518107, China; State Key Laboratory of Biocontrol, School of Ecology, Sun Yat-sen University, Shenzhen 518107, China

**Keywords:** *Charadrius*, long-read sequencing, speciation genomics, local adaptation, shorebirds

## Abstract

The *Charadrius* genus encompasses 36 species, each exhibiting a wide array of life histories and behaviors. Conspecific variations in mating systems and migratory behavior also exist in widely distributed species, such as the Kentish plover (*Charadrius alexandrinus*). Recent years have witnessed the establishment of new species from distinct populations, such as the identification of the snowy plover (*C. nivosus*) and the white-faced plover (*C. dealbatus*) as separate species from the Kentish plover (*C. alexandrinus*) species complex. Studies on the phylogenetic relationships across the entire genus, as well as population genetics and adaptation to various climatic conditions, necessitate the support of a high-quality reference genome. Here, we successfully assembled the first high-quality genome for the white-faced plover. The genome size is 1.25 Gb, anchored on 31 chromosomes, with a contig-level N50 size of 23.62 Mb and a scaffold-level N50 of 84.07 Mb. The completeness of the *C. dealbatus* genome assembly is supported by the 97.3% completeness (BUSCO) and ultraconserved element (UCE) (4,889/5,041; 97.0%) loci retrieved. A total of 17,641 protein-coding genes were predicted among 19,648 pseudogenes, and the completeness of the genome annotation is 94.7% (BUSCO). The chromosome-level genome provides the availability of permanent genomic resources for advancing evolutionary and conservation genomic studies on the genus *Charadrius*.

Significance This contiguous, accurate, and annotated genome assembly represents a significant resource for eco-evolutionary investigations focused on speciation, adaptation, and conservation in wild populations. It provides a significant reference for the genus *Charadrius*, which is crucial for advancing our understanding of local adaptation and colonization history across continents, particularly given the current limited genomic resources for the species.

## Introduction

*Charadrius* plovers, belonging to the Charadriidae family, represent a diverse group of shorebirds characterized by a range of morphological traits, ecological behaviors, and life histories (Conklin 2019). The genus *Charadrius* traces its origins back to the Neogene period, marked by a rapid diversification that resulted in the emergence of 36 distinct lineages over 10 to 15 million years (Stiller et al. 2024).

Previous studies on the phylogenetic relationships within *Charadrius*, primarily based on mitochondrial DNA and nuclear microsatellite markers, suggest a paraphyletic pattern (Dos Remedios et al. 2015; Černý and Natale 2022). Genomic data presents a promising avenue for addressing these challenges, particularly in elucidating the demographic histories and genome-wide divergence patterns of emerging shorebirds (Zhou et al. 2024). However, frequent observations of chromosome rearrangements in Charadriiformes (Hill et al. 2023; Sozzoni et al. 2023) suggest that such alterations likely hinder gene flow (Mackintosh et al. 2023). The intricate process of speciation with ongoing gene flow persists within *Charadrius*. To determine whether structural variants contribute to hybrid fitness and reproductive isolation in *Charadrius*, a thorough resolution requires high-quality chromosome-level reference genomes. Furthermore, considering the diverse range of *Charadrius* species occupying heterogeneous and specialized habitats, such as the Kentish plover (*C. alexandrinus*) populations inhabiting high plateaus and coastal beaches (Mcdonald et al. 2022), as well as displaying migratory or resident behaviors, exemplified by the little ringed plover (*C. dubius*) (Hedenström et al. 2013), it becomes paramount to investigate their adaptive mechanisms within various environments from a microevolutionary standpoint. The potential of long reads is promising in delineating the genetic architecture and haplotype information associated with adaptive traits, particularly in the context of selective sweeps (Bock et al. 2023).

Moreover, there are small and threatened populations within the *Charadrius* genus that require immediate attention. For instance, the endangered Madagascar plover (*C. thoracicus*) is estimated to have less than 3,500 individuals (Zefania et al. 2008). Similarly, the St. Helena plover (*C. sanctaehelenae*) had a population of fewer than 560 individuals in 2019 (BirdLife International 2024, https://datazone.birdlife.org/species/factsheet/saint-helena-plover-charadrius-sanctaehelenae). Given their small population sizes, it is imperative to assess their genetic health due to the presence of detrimental effects of genetic load for planning a genetic management project (e.g., Chen et al. 2023). The availability of a reference genome therefore offers a comprehensive resource of the entire genome and can prove immensely beneficial for the conservation efforts aimed at safeguarding these vulnerable plover species.

In our research, we reported the first chromosome-level genome assembly for the white-faced plover (C. *dealbatus*), which diverged from the Kentish plover less than half a million years ago (Wang et al. 2019; Zhou et al. 2024). This assembly is accompanied by a comprehensive description of its genetic makeup, which aims to serve as a valuable genomic resource within the *Charadrius* genus. This resource is to facilitate in-depth investigations into topics such as speciation, local adaptation, and conservation genomics across different populations and species within the *Charadrius* genus.

## Results and Discussion

### Genome Assembly Features and Quality

**Fig. 1.**
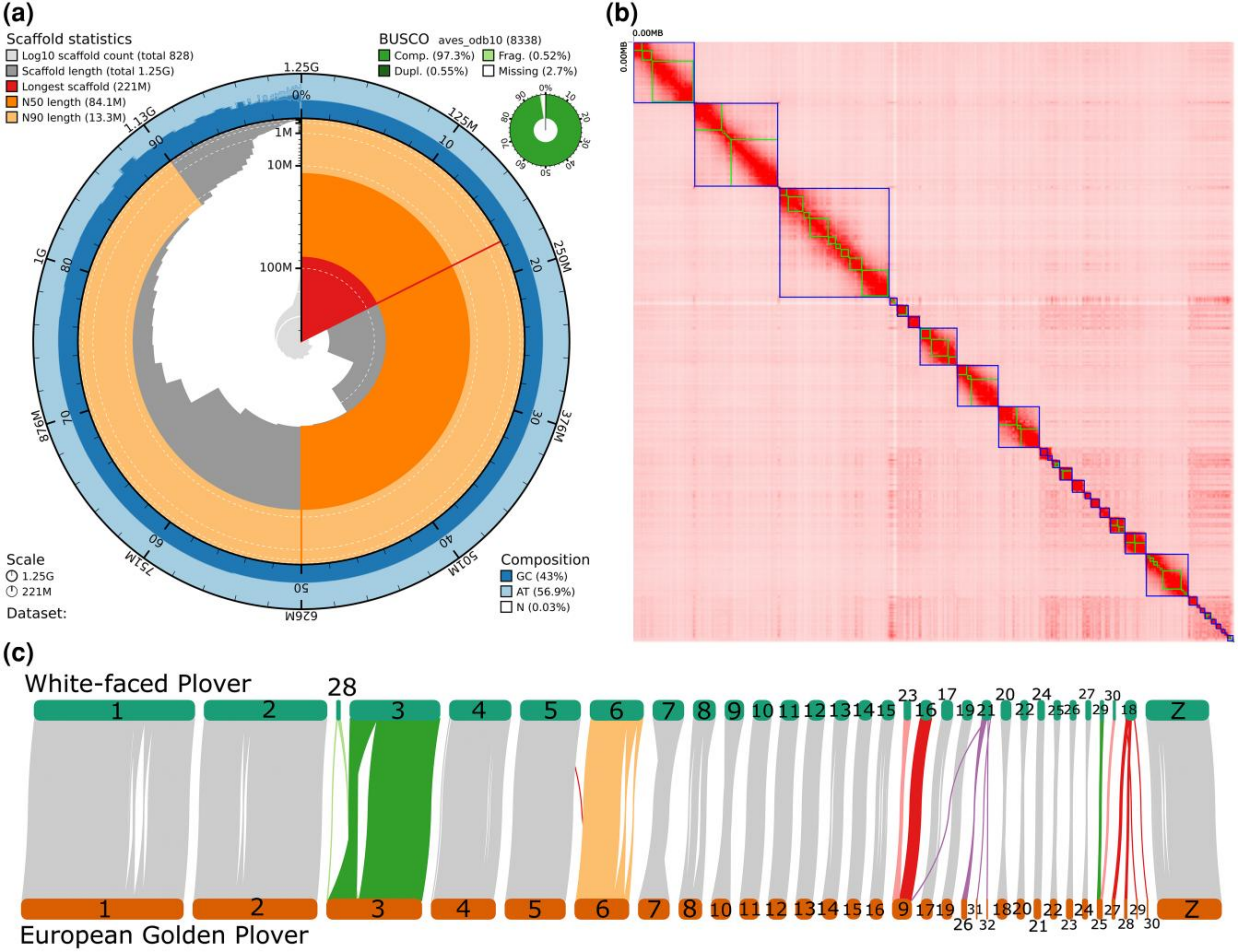
a) Snail plot shows results of scaffold statistics, BUSCO, and the composition of the chromosome-level genome of *C. dealbatus*. b) Hi-C interaction map of the chromosome-level genome of *C. dealbatus*. The plot was drawn in 50 Mb bin size. The stronger the signal, the stronger the interaction and the closer the distance. Blue represents chromosome boundaries, with each blue box indicating a chromosome; green represents contig boundaries, with each green box indicating a contig. c) Pairwise whole-genome alignments between white-faced plover (*C. dealbatus*, top) and European golden plover (*P. apricaria*, bottom). Each horizontal bar represents a different chromosome. The numbers in the bars show the chromosome IDs of each species. The results showed 3 fusion events (white-faced plover chr28 + chr3, chr23 + chr16, and chr29 + chr30) and 2 fission events (white-faced plover chr21 and chr18).

The assembled primary genome using 144 Gb PacBio long reads consists of 828 scaffolds (scaffold N50 of 84.07 Mb) and 1,206 contigs (contig N50 of 23.62 Mb), with a total length of 1.25 Gb (Fig. 1). Compared to the previous assembly of Kentish plover (*C. alexandrinus*) using short-read data, the whole-genome quality of white-faced plover is significantly higher (Table 1). It is also comparable with the counterpart of the European golden plover (*Pluvialis apricaria*) using long-read data (Table 1). A total of 96.78% of Illumina short reads can be properly mapped to the assembled genome, and 97.3% of Benchmarking Universal Single-Copy Orthologs (BUSCO) sequences for plover were complete (Table 1), showing excellent completeness and accuracy of the genome. In detail, 8067 single-copy genes (96.7%), 46 completely duplicated genes (0.6%), 43 fragmented genes (0.5%), and 182 missing genes (2.2%) were identified (Fig. 1). It is comparable with the telomere-to-telomere genome of chicken (GCA_024206055.2), 97% of BUSCO scores.

**Table 1:** Comparison of assembly statistics, BUSCO results, and annotation statistics between the *C. dealbatus* (this assembly) and *P. apricaria* genome assemblies

Assembly statistics	*C. dealbatus*	*P. apricaria* (bPluApr1.pri)
Assembly size (Gb)	1.25	1.25
Number of contigs	1206	374
Contigs N50 (Mb)	23.62	15.2
Contig L50	14	23
Maximum contig size (Mb)	94.85	79.61
Number of scaffolds	828	106
Scaffold N50 (Mb)	84.07	87.5
Scaffold L50	5	5
Maximum scaffold size (Mb)	220.66	222.69
Number of scaffolds >50 kb	185	50
Number of chromosomes	31	32
BUSCO assembly (aves_odb10)	C: 97.3% (S: 96.7% D: 0.6%), F: 0.5%, M: 2.2%	/
BUSCO annotation (aves_odb10)	C: 94.7% (S: 94.1% D: 0.6%), F: 4.5%, M: 0.8%	/
Genes and pseudogenes	19,648	…
Predicted protein-coding genes	17,641	/

The genome assembly is composed of 31 chromosomes (30 autosomes and 1 Z chromosome), without signal of the W chromosome as the Hi-C sequencing was performed on a male individual. While our Hi-C map appears noisy, this reflects the suboptimal library's quality, which yielded a low cis/trans ratio of approximately 0.47 and a low number of valid read pairs (Table S5) (Oksuz et al. 2021), rather than a flaw in the scaffolding process. The high quality of our genome assembly is supported by multiple pieces of evidence. At the contig level, the assembly is relatively free of contamination due to the taxonomic consistency of our assembly (Fig. S1). Furthermore, the BUSCO assessment of the 31 putative chromosomes revealed a high completeness of 98.4% (Fig. S2), which represented 96.28% of the total assembly. Despite the noise appearing in the Hi-C map, the clear block-diagonal pattern indicates successful scaffolding from the assembly. The remaining 154 unplaced scaffolds longer than 50 kb likely represent microchromosomes or other genomic fragments that could not be confidently anchored to the main chromosomes.

### Genome Synteny

Our final white-faced plover genome of 1.25 Gb mapped on 75.26% of the closest reference genome available (*P. apricaria*, 1.2 Gb), a species from which the *Charadrius* diverged in the late Cretaceous (Baker et al. 2007). The 31 pseudochromosomes of *C. dealbatus* assembly mapped on all 33 chromosomes of the *P. apricaria* (Fig. 1c). We observed 5 interchromosomal rearrangement events between the 2 species, including 3 fusion events (*C. dealbatus* chr28 + chr3, chr23 + chr16, chr29 + chr30) and 2 fission events (*C. dealbatus* chr21, chr18). These rearrangement events are distinct from the synteny between the European golden plover and the other 3 species (*Rissa tridactyla*, *Sterna hirundo*, and *Alca torda*) in Charadriiformes (Sozzoni et al. 2023), which suggests most rearrangements are lineage-specific in Charadriiformes.

### Genome Annotation

The GC content of the final genome was 43.05%. All repetitive elements account for 10.72% of the total length of the genome, with interspersed repeats accounting for 9.58%, including 7.93% retroelements, 0.12% DNA transposons, and 1.53% unclassified repeat sequences. Tandem repeats, including simple repeat sequences and satellite sequences, account for 1.13%. We identified 19,648 genes and pseudogenes in the genome, including 17,641 protein-coding genes, and the BUSCO completeness was 94.7%, with a 0.6% duplication rate, 4.5% fragment rate, and 0.8% missing rate.

## Materials and Methods

### Sample Collection and Sequencing

White-faced plover samples were collected from Paisha Island, in Zhanjiang, Guangdong Province, China, between 2019 and 2022. Fresh blood was used for short-read DNA sequencing from a female individual. Breast muscle was used for continuous long-read sequencing from a dying female plover accidentally sampled. A broken egg with a dead embryo caused by predation was flash-frozen in liquid nitrogen for Hi-C library construction. According to our subsequent detection (Table S1), the fractured egg was a male, leading to the W chromosome being unassembled. Iso-Seq from the fresh blood of 10 plovers was used for the full-length transcriptome sequencing to facilitate functional gene annotation. All samples were kept at −80 °C before being processed.

Short reads DNA was extracted by using the Qiagen DNeasy Blood and Tissue kit (Qiagen, United States) and quantified for quality using Agilent 5400 (Agilent Technologies, California, United States). One pair-end library was constructed with insert sizes around 150 bp and sequenced on the Illumina HiSeq X platform. The Hi-C library was constructed using the *MboI* restriction enzyme and sequenced on the Illumina HiSeq platform. Long-read DNA was extracted by using the Qiagen DNeasy Blood and Tissue kit (Qiagen, GmbH, Hilden, Germany) according to the manufacturer's protocol. One 20 kb HiFi SMRTbell library was constructed according to PacBio's protocol and sequenced on the Pacific Biosciences (PacBio) Sequel single-molecule real-time (SMRT) platform (PacBio RSII) in the Genome Center of Novo Genomics (Novogene, Beijing, China). RNA was extracted using TRIzol reagent (Thermo Fisher Scientific) according to the manufacturer's instructions, then detected for purity by NanoDrop, completeness by Agilent 2100 (Agilent Technologies, California, United States), and finally quantified by Qubit 2.0. One RNA library was constructed and sequenced on the Illumina NovaSeq 6000 platform.

### Assembly Pipeline

To obtain quality sequencing data, we first filtered out adapter sequences and low-quality reads. Then we assembled PacBio long reads to a draft genome by Wtdbg2 v2.5 (Ruan and Li 2020) following arguments: -K 2000 --edge-min 4 -p 17 -S 4 -L 5000 --tidy-reads 8000. Next, we performed a draft genome polish with mainly 2 steps: mapping back our PacBio long reads by Minimap2 v2.17 (Li 2018) and Samtools v1.9 (Mcdonald et al. 2022) and combining with short-read DNA iteratively polished twice by NextPolish v1.4.1 (Hu et al. 2020). Subsequently, we scaffolded the draft genome with the Hi-C reads using 3D-DNA v180922 (Dudchenko et al. 2017). Prior to this, Hi-C reads were mapped by Bwa v0.7.17 (Li and Durbin 2009) and processed with Juicer v1.6 (Durand et al. 2016b) to identify potential enzyme cutting sites. Finally, we adjusted the genome manually with Juicebox v1.11.08 (Durand et al. 2016a).

### Gene Annotation

Both ab initio and homology-based methods were employed to identify repetitive elements in genome assembly. For the homolog-based method, RMBlast v2.13.0 was used to search against the Repbase library, and RepeatMasker (www.repeatmasker.org; last accessed 2019 November 22) was applied for annotation. In the ab initio method, a custom repeat library was generated using RepeatModeler (www.repeatmasker.org) and subsequently utilized in RepeatMasker for repeat annotation.

To annotate protein-coding genes, we processed all Iso-Seq reads using the official Iso-Seq pipeline (https://isoseq.how/clustering/cli-workflow.html). First, primers were removed, poly(A) tails were trimmed, and rapid concatemer identification and removal were performed. The cleaned reads were then clustered and converted into a FASTA file using Samtools v1.9 (Danecek et al. 2021). Gene structure prediction was conducted using the MAKER v3.01.04 pipeline (Cantarel et al. 2008), which integrates 3 approaches: ab initio prediction, transcriptome-based evidence, and homology-based prediction.

For ab initio gene prediction, we utilized Augustus v3.4.0 (Stanke and Morgenstern 2005), BRAKER v2.1.6 (Kollmar 2019), GeneMark-ET v3.62 (Lomsadze et al. 2014), and SNAP v2.51.7 (Leskovec and Sosič 2016) to generate gene models. Transcriptome-based predictions were obtained using Exonerate. For homology-based prediction, protein sequences from 13 different avian species were incorporated: *Aramus guarauna* (GenBank accession: GCA_013400195.1), *Balearica regulorum* (GCA_000709895.1), *Charadrius vociferus* (GCA_000708025.2), *Columba livia* (GCA_000337935.2), *Cuculus canorus* (GCA_000709325.1), *Grus americana* (GCA_013390085.1), *Grus nigricollis* (GCA_004360235.1), *Heliornis fulica* (GCA_013399135.1), *Opisthocomus hoazin* (GCA_000692075.1), *Psophia crepitans* (GCA_013399095.1), *Spheniscus magellanicus* (GCA_010076225.1), *Taeniopygia guttata* (GCF_008822105.2), and *Gallus gallus* (GCA_000002315.5).

Finally, we merged the outputs from 3 prediction approaches using MAKER's default settings to generate weighted consensus gene structures. Protein sequences were extracted using a Perl script (Christiansen et al. 2012), and functional annotation was performed using Blastp (Li and Lu 2019) and InterProScan v5 (Jones et al. 2014).

### Genome Quality and Completeness Assessment

To assess the completeness of genome assembly and annotation, the results were compared to the BUSCO based on 8338 universal avian single-copy orthologs (aves_odb10) (Waterhouse et al. 2013). We also analyzed the statistics results (Table S2) to assess consistency between short reads and long-read assembly, following mapping the Illumina short reads of *C. dealbatus* (SYSb005956) to the chromosome-level genome of *C. dealbatus*. A snail plot was generated using BlobToolKit (Challis et al. 2020 ) to visualize the assembled genome quality.

### Syntenic Estimates

To compare white-faced plover assembly to previously available species assembly (European gold plover, bPluApr1.pri), we aligned 2 genomes using nucmer (-b 400) in the MUMmer package (v4.0.0rc1) (Marçais et al. 2018). We kept only 1-to-1 alignments by delta-filter (−1) in number, removing the scaffolds that were not assigned to chromosomes. Pairwise alignments were then formatted for input into the NGenomeSyn pipeline for synteny visualization (He et al. 2023).

## Supplementary Material

evaf219_Supplementary_Data

## Data Availability

The final genome assembly and annotation (*C. dealbatus*) are available under the NCBI (accession number: JBQZFF000000000).
