# Supplementary material for: A Chromosome-Level Reference Assembly and Annotation for the White-Faced Plover (Charadrius dealbatus) Genome
Source: Genome Biol Evol. 2025 Dec 3;17(12):evaf219. doi: 10.1093/gbe/evaf219 (PMC12708339; doi:10.1093/gbe/evaf219)
Supplement: evaf219_Supplementary_Data [file evaf219_supplementary_data.docx]

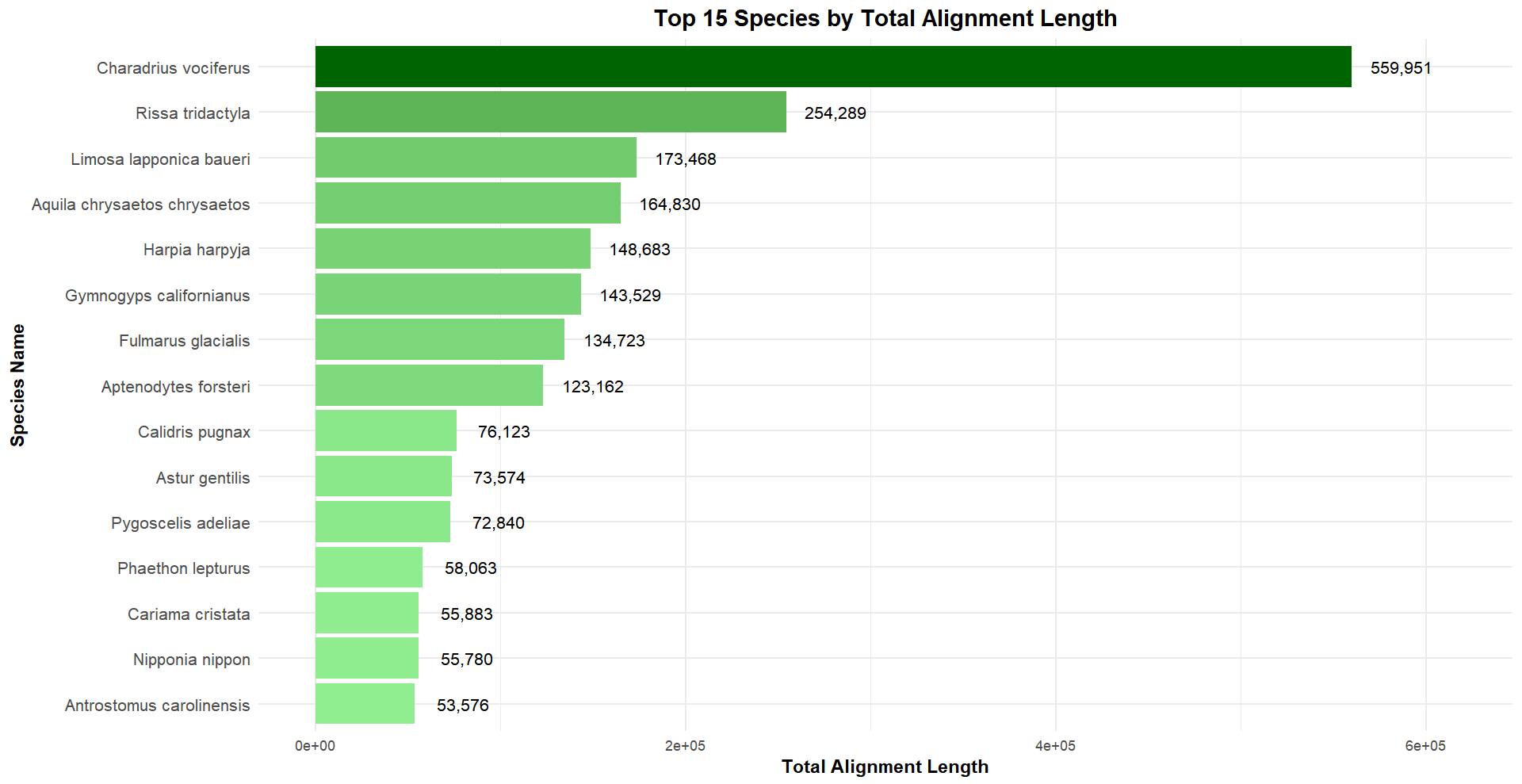


Figure S1. Top 15 species based on alignment length from BLAST results of the contig-level genome against the NCBI nr database.


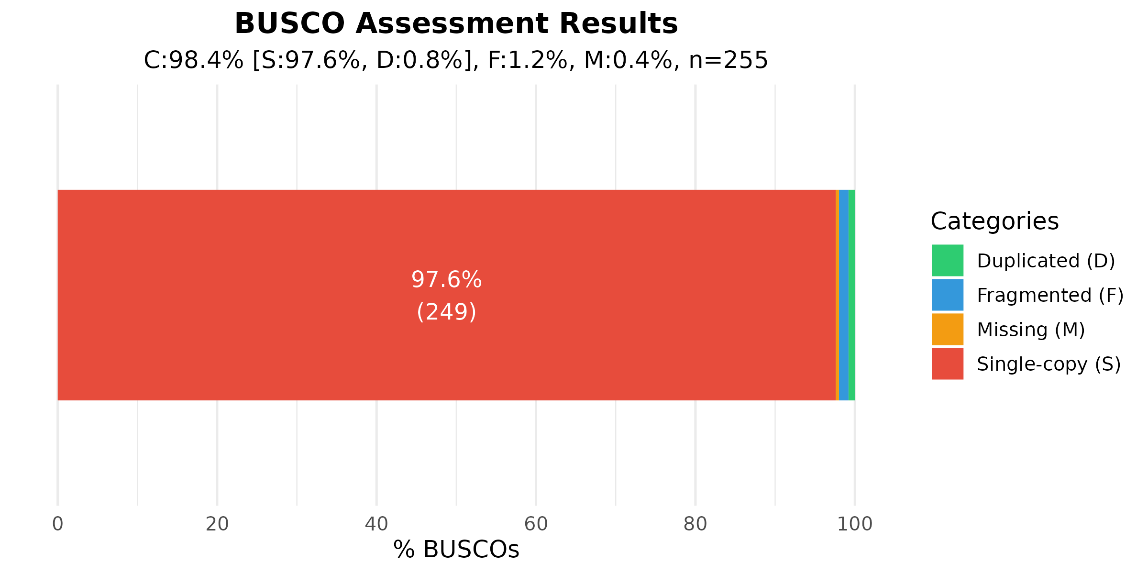


Figure S2. The BUSCO scores of the given 31 chromosomes.

Table S1. Hi-C sequences mapping with the sexual chromosomes of European Golden Plover (*Pluvialis apricaria*).

| **Name** | **startpos** | **endpos** | **numreads** | **covbases** | **coverage** | **meandepth** | **meanbase** | **meanmapq** |
| --- | --- | --- | --- | --- | --- | --- | --- | --- |
| W | 1 | 32002124 | 9775566 | 11181698 | 34.94 | 34.45 | 35.9 | 6.4 |
| Z | 1 | 87527607 | 36959081 | 81115859 | 92.67 | 50.44 | 35.8 | 51.6 |

Table S2. Statistics results of short-read mapping with chromosome-level genome using Samtools.

|  | **details** | **results** |
| --- | --- | --- |
| Illumina read mapping | Total reads  Mapping rate | 122073920  99.69% |
| Samtools statistics | Properly paired | 96.78% |
|  | singletons | 0.01% |
|  | duplicates | 0 |
|  | supplementary | 0 |
|  | secondary | 0.03% |

Table S3. Summary of short-read sequencing data used by this study after quality control.

| **Library type** | **Clean data (Gbp)** |
| --- | --- |
| Whole genome short gun | 3.75 |
| RNA short-read library (blood) | 1.28 |

Table S4. Summary statistics of the *Charadrius dealbatus* genome annotation repetitive element.

| **Repetitive element** | Total | 10.72% |
| --- | --- | --- |
|  | Interspersed repeats | 9.58% |
|  | Retroelements | 7.93% |
|  | DNA transposons | 0.12% |
|  | Unclassified | 1.53% |
|  | Other (Satellites, Simple repeats, Low complexity) | 1.13% |

Table S5. Detailed quality control data of Hi-C sequencing.

|  | Read 1 (bp) | Read 2 (bp) | Di-Tag (bp) |
| --- | --- | --- | --- |
| Total Reads | 9531333 | 9531333 |  |
| Failed To Align | 1363068 | 1469020 |  |
| Valid Pairs |  |  | 4291610 |
| Invalid Pairs |  |  | 1252125 |
| Cis-Pairs |  |  | 1376580 |
| Trans-Pairs |  |  | 2915030 |
